# The lipidome of an omnivorous insect responds to diet composition and social environment

**DOI:** 10.1002/ece3.9497

**Published:** 2022-11-08

**Authors:** Yeisson Gutiérrez, Marion Fresch, Christoph Scherber, Jens Brockmeyer

**Affiliations:** ^1^ Centro de Bioinformática y Biología Computacional de Colombia – BIOS Manizales Colombia; ^2^ Institute for Biochemistry and Technical Biochemistry University of Stuttgart Stuttgart Germany; ^3^ Institute of Landscape Ecology University of Münster Münster Germany; ^4^ Centre for Biodiversity Monitoring Zoological Research Museum Alexander Koenig Bonn Germany

**Keywords:** *Acheta domesticus*, differential expression, flow injection analysis, house cricket, lipidomics, liquid chromatography, MS/MS ALL, multifactorial experiment, nutritional ecology, tandem mass spectrometry

## Abstract

Lipids are biomolecules with essential roles in metabolic processes, signaling, and cellular architecture. In this study, we investigated changes in the lipidome of the house cricket *Acheta domesticus* subjected to diets of different nutritional composition (i.e., protein to carbohydrate ratio) and two distinct social environments (i.e., solitary or in groups). We measured relative abundances of 811 lipid species in whole‐body cricket samples using flow injection analysis coupled to tandem mass spectrometry. We assessed differences in the relative abundances of lipid species induced by diet composition and social environment in female and male *A. domesticus*. Additionally, we performed a functional analysis of the lipids with significant differences using a recently developed database. We found that most differences in the relative abundances of lipid species were explained by sex alone. Furthermore, the lipidome of female *A. domesticus* was responsive to diet composition. Females fed with the balanced diet had an even higher abundance of lipids involved in lipid storage than their counterparts fed with a protein‐rich diet. Interestingly, the male cricket lipidome was not responsive to diet composition. In addition, the social environment did not induce significant changes in the lipid profile neither in female nor in male crickets.

## Graphical abstract

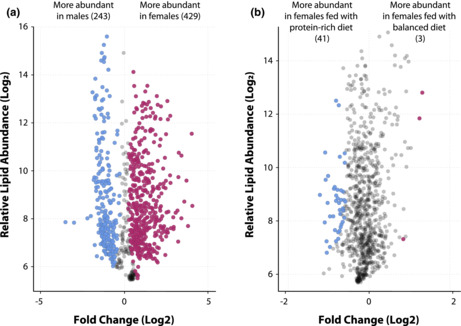

## 1 INTRODUCTION

Lipids are a heterogeneous group of compounds with essential roles in metabolic processes (i.e., energy production and storage), signaling, cellular architecture, and functioning of the cellular membranes (Fahy et al., 2007; Jones et al., 2004; Mertens et al., 2017; Shevchenko & Simons, 2010). Contrary to several other biomolecules studied with “omics” techniques (e.g., RNA transcripts and proteins), lipids are produced and metabolized through enzymatic activity (i.e., they are not genetically encoded) (Nguyen et al., 2017; Wenk, 2010). The analysis of the lipid composition of a cell, tissue, or organism is termed lipidomics. This discipline aims to the description and quantitation of lipid species, their biological functions, and understanding of the implications of lipid diversity (Jones et al., 2004; O'Donnell et al., 2020; Shevchenko & Simons, 2010).

Lipidomics is considered an emerging field mainly driven by human medicine as many drugs target lipid metabolism and signaling pathways (Wang et al., 2016; Wenk, 2010). Yet, the thorough study of lipid profiles has also proven to be informative for the understanding of invertebrate physiology (Colinet et al., 2016; Martin et al., 2019). Moreover, previous studies have demonstrated that lipidomics bear a great potential to unravel physiological changes in insect responses to environmental changes (Colinet et al., 2016; Hayward, 2014). And, in some cases, analyses of the lipidome were particularly useful to investigate short‐term (i.e., a few days) responses to changing environments (Colinet et al., 2016). A considerable amount of literature has reported that the phenotype (i.e., morphological and behavioral traits) of an organism can be greatly influenced by challenging environments (e.g., Callahan et al., 1997; Gabriel, 2005; Gutiérrez, Ott, et al., 2020; Gutiérrez, Phung, et al., 2020; Schulte, 2014). Yet, the underlying physiological mechanisms of the phenotypic plasticity exhibited in response to changes in environmental factors are largely unknown. Therefore, the detailed study of the alterations in the profile of biomolecules with meaningful biological roles, such as lipids, can be informative to infer the physiological mechanisms driving changes of the phenotype (Dennis, 2009).

Previous studies have shown that the lipidome of several taxa is responsive to seasonal changes (Koštál et al., 2013), organic pollution (Li et al., 2020; Rocchetta et al., 2014), microbial infection (Gołębiowski et al., 2020), parasitism (Wang et al., 2020), diet (Carvalho et al., 2012; Kazek et al., 2019; Meyer et al., 2020), and housing conditions (Sato et al., 2013). Yet, the research to date has concentrated on single‐factor analysis as the multifactorial analysis of the “omics” dataset has been problematic in the past as interaction terms ought to be included in the statistical modeling (Guisset et al., 2019; Lambert et al., 2020). Additionally, many studies have focused on the comparison of lipid amounts due to the lack of functional‐annotation tools, which has limited the potential of these analyses (Nguyen et al., 2018; O'Donnell et al., 2020). In this study, we take advantage of a robust mass spectrometry‐based technique to quantify the lipidome of an entire organism (Simons et al., 2012), a novel multifactorial analysis approach (Čuklina et al., 2018), including a recently developed functional annotation tool for lipidomics datasets (Molenaar et al., 2019).

Our model organism, the house cricket *Acheta domesticus* (L., 1758), is a cosmopolitan species widely used for behavioral and physiological studies (e.g. Crocker & Hunter, 2018; Gray, 1997; Nosil, 2002), and mass‐produced as food and feed (Oppert et al., 2020; Udomsil et al., 2019). In this insect species, the total lipid content accounts for 10% of the dry weight (Udomsil et al., 2019) and 5–7% of the fresh weight (Gutiérrez, Fresch, et al., 2020) of the body composition. To the extent of our knowledge, studies on the lipidome of *A. domesticus* have mainly addressed general lipid composition in a descriptive way (Grapes et al., 1989; Hutchins & Martin, 1968; Lambremont & Dial, 1980; Martin & Carls, 1968; Wang & Patton, 1969) and developmental changes in some lipid categories and classes (Cripps et al., 1988; Cripps & De Renobales, 1988). However, no attempt has been made to examine the changes in the lipid profile induced by distinct diets and social environments in the house cricket *A. domesticus*.

Here, we studied the lipid profile of crickets subjected to contrasting experimental conditions (i.e., fed with either of two diets differing in nutritional composition and housed either solitarily or in groups). Our aim was to elucidate physiological changes related with changes in the lipidome of the house cricket *A. domesticus*. We hypothesized that: (1) there will be significant differences in the lipidome of male and female crickets related to sex‐specific traits and distinct life strategies. (2) Females living in groups will evidence greater lipid storage than their counterparts living solitarily as suggested by previous studies (e.g. Gutiérrez, Fresch, et al., 2020). (3) Females fed with the protein‐rich diet, which achieved a higher fecundity in a previous study (Gutiérrez, Fresch, et al., 2020), will exhibit a higher abundance of lipids related to storage function as lipids are the main energy source for the developing embryo (Van Handel, 1993; Ziegler & Van Antwerpen, 2006).

## 2 MATERIALS AND METHODS

### 2.1 Experimental design and sample collection

In this study, we used samples collected from a recent experiment (Gutiérrez, Fresch, et al., 2020) in which we subjected the house cricket *A. domesticus* (L. 1758) to distinct experimental conditions. We manipulated the diet composition (diets contained either 1:1 or 3:1 protein to carbohydrate ratio) and the social environment (insects reared either in solitarity during their whole life cycle or at a density of 800 inds m^−2^ – i.e., six individuals in every container). Such dissimilar conditions were implemented aiming to track resource allocation and investment in a model omnivorous species subjected to contrasting experimental treatments. For further details on the experiment setup and insect husbandry, see Gutiérrez, Fresch, et al. (2020). We studied the lipidome of the house cricket *A. domesticus* following a three‐factor experimental design (i.e., Diet × Social Environment × Sex) with eight resulting experimental treatments. We collected five biological replicates (i.e., whole insects) for every experimental treatment (*N* = 40, Table 1). All insects were collected 15 days after the adult molt to avoid introducing confounding factors caused by age‐related physiological differences (Cripps et al., 1988). All insects were alive at the time of collection and immediately frozen at −20°C.

**TABLE 1:** Summary of the experimental design and sample size

Factor 1: diet composition	Factor 2: social environment	Factor 3: sex	Biological samples (i.e., insects)
1:1	Solitary	Male	5
1:1	Solitary	Female	5
1:1	Group	Male	5
1:1	Group	Female	5
3:1	Solitary	Male	5
3:1	Solitary	Female	5
3:1	Group	Male	5
3:1	Group	Female	5

*Note*: Diets contained either 1:1 or 3:1 protein to carbohydrate ratio. Social environments consisted in insects reared either in solitarity during their whole life cycle or at a density of 800 inds m^−2^ – i.e., six individuals in every container.

### 2.2 Sample preparation

The protocol explained below was used to process both insects and diet samples in the same manner. Extraction of lipids was done following the method of Folch et al. (1957), with some modifications (see Figure S1 in the Appendix S1). Insects were individually homogenized with an Ultra‐Turrax Tube Drive (IKA®‐Werke GmbH & Co., Germany) in methanol (1 + 30 w/v) at 6000 rpm during 3 min. Afterward, two technical replicates were collected from every homogenate and a two‐fold 1:50 (v/v) dilution was achieved. Subsequently, we added chloroform to reach a 2:1 (v/v) cloroform:methanol solution in specific extraction tubes, and a mixture of 13 deuterium‐labeled lipids covering all lipid classes was added as the internal standard, and every lipid concentration was 100 μg/ml (EquiSPLASH™ Lipidomix®, Avanti Polar Lipids, Inc.) (see Table S1 in the Appendix S1). Afterward, the lipids were extracted by shaking vigorously in the cold for 1 h at 200 U/min. A volume of 1.2 ml of 150 mM ammonium bicarbonate was added to force polar lipids into the organic phase, and the solution was shaken for 10 more minutes. Thereafter, samples were centrifuged at 5000 rpm during 10 min at 4°C (Z 326 K, Hermle Labortechnik GmbH), and the bottom organic phase was stored in glass vials. The chloroform of the organic phase was evaporated in a vacuum concentrator at 30°C and 1500 rpm (RVC 2‐18 CDplus, Martin Christ Gefriertrocknungsanlagen GmbH). Finally, the lipids were dissolved in 150 μl of 10 mM ammonium acetate in methanol, and the solution was transferred to glass micro‐inserts. All insect (80 = 40 biological replicates × two technical replicates) and diet (4 = 2 replicates of each diet type) samples were then stored at −80°C until further measurement. A graphical summary of the extraction protocol is available in Figure S1 in the Appendix S1. All reagents for lipid extraction and FIA‐MS/MS analysis (below) were obtained from Merck KgaA (Germany), unless otherwise stated.

### 2.3 Sample analysis (FIA‐MS/MS)

For the detection and quantification of lipids, we used a data‐independent approach termed MS/MS^ALL^ in the Analyst TF 1.8 software (Sciex), which allows for acquisition of fragment ion spectra from all precursors within a defined mass range. A volume of 50 μl of sample was injected into a TripleTOF™ 6600 mass spectrometer (Sciex) equipped with a 50 μm (internal diameter) electrospray‐ionization (ESI) electrode. Injection was performed through flow injection analysis (FIA) by means of a M3 Micro LC (Sciex), and the mobile phase consisted of a solution of 2 mM ammonium acetate in methanol. The flow rate was set to 4 μl/min for 6.5 min and increased to 50 μl/min for 1.5 min to wash the system before equilibration at 4 μl/min for 2 min prior to the next run. MS acquisition started 1 min after sample injection and lasted for c. 5 min. Detailed MS parameters are available in Table S2 in the Appendix S1. All 80 insect samples (40 biological replicates × two technical replicates) and the four diet samples (two diets × two biological replicates) were measured consecutively in random order with the same instrument in both positive and negative ion mode for full lipid coverage. We performed a calibration run every seven samples using ESI calibration solutions (Sciex) specific for either positive or negative ion mode.

Mass spectrometry (MS/MS) data were processed using the LipidView 1.3 beta software (Sciex). Lipid species were identified through four database searches, one for each sample type (cricket or diet) and acquisition mode (i.e., positive, or negative). Concerning the cricket samples, eight MS data files were used for lipid identification (one representative sample from every experimental treatment was randomly chosen), while two MS data files were used for diet samples (one of each diet, Table S3 in the Appendix S1). Targeted methods containing the characterized lipid species with their corresponding precursor and fragment ion mass‐to‐charge ratios as well as isotope correction factors were then created for each of those four cases by retaining the most abundant lipid species. Precursor and fragment ions of each internal standard were also included in those methods and assigned to each targeted lipid species depending on the lipid class (see Table S4 in the Appendix S1). As the lipids herein reported were identified through tandem MS, they can be regarded as level 1, non‐novel metabolite identification (Sumner et al., 2007). Moreover, using MS/MS spectra allows confirming the headgroup, the constituent fatty acyls (the fatty acyl/alkyl level), and the regioisomeric positions (the fatty acyl/alkyl position level) (Züllig et al., 2020). Therefore, the nomenclature herein used is compatible with current efforts to standardize reporting standards.

All MS result data files were finally processed with the corresponding targeted method for the calculation of the lipid fragment ion peak areas (i.e., relative amounts) as follows:

$$\text{Corrected area}=\frac{{\text{Area}}_{\text{analyte}}\times {\text{IsotopeCorr}}_{\text{analyte}}\times {\text{Amount}}_{\text{IS}}}{{\text{Area}}_{\text{IS}}\times {\text{IsotopeCorr}}_{\text{IS}}\times {\text{Amount}}_{\text{analyte}}}$$

Areas of the analyte and internal standard (IS) fragment ion peaks were calculated by the software. IsotopeCorr is the correction for isotopic peak distribution for a specific lipid species. Amount_IS_ was set to 100, and Amount_analyte_ received a default value of 1. Internal standard for correction was assigned depending on the lipid class.

### 2.4 Data analysis

The lipid species identified and quantified in the positive (380) and negative (445) ion modes were combined into a single spreadsheet (for insects and diets separately). From the total lipid species contained in the insect dataset, 14 were identified in both modes, for which the highest intensities were retained (all of them from the negative ion mode). Peak areas of the technical replicates were averaged (the Pearson correlation coefficient between technical replicates was >0.95 in all cases). Therefore, the final insect dataset contained the relative abundance of 811 lipid species for the 40 biological samples. Yet, one biological sample (a female cricket from the treatment: Diet 3:1 and living in solitary) had to be excluded from the insect dataset because of evident anomalies in the lipid profile after data acquisition likely due to problems during sample preparation (see the raw data in the Appendix S1).

The sample medians were centered to the global median of the whole dataset using the library proBatch (‘normalize_sample_medians_dm[]’ function) (Čuklina et al., 2018) (see Figure S2 in the Appendix S1). Groups of seven samples between calibration runs were considered batches. Subsequently, exploratory data analysis (principal component analysis and hierarchical clustering heatmap) was done using the proBatch (‘plot_heatmap_diagnostic’ function) (Čuklina et al., 2018) and factoextra libraries (‘prcomp’ and ‘fviz_pca_ind’ functions) (Kassambara & Mundt, 2017). A multivariate analysis of variance (MANOVA) was also used to explore differences among experimental treatments including the first three principal components (from the PCA analysis, which explained 85.95% of variances) as dependent variables. Subsequently, pairwise comparisons among the eight experimental treatments (i.e., diet × social environment × sex) were conducted using a Wilks' lambda test and Bonferroni correction with the library biotools (Da Silva, 2015).

Differences in the relative abundance of lipid species among experimental treatments were assessed using the tool DiCoExpress (Lambert et al., 2020). This tool integrates functions from the package edgeR (Robinson et al., 2010) originally intended for multifactorial analysis of gene expression data, but the principle is widely applicable to other “omics” data (Langley & Mayr, 2015). Initially, differences in the abundance of lipids were tested by mean contrasts inside generalized linear models where the experimental factors (described above) and the interaction of them were included in the models. The *p*‐values were adjusted by the Benjamini–Hochberg procedure, and an alpha (FDR) of .05 was considered a threshold for significance.

Afterward, functional annotation (i.e. lipid ontology) of the lipid species was done using the the LION/web tool (Molenaar et al., 2019). First, lipid names were standardized according to Lipid Maps nomenclature (Fahy et al., 2009) via RefMet Metabolomics Workbench. Posteriorly, the Lipid Ontology (LION) terms for the domains, lipid classification, biological function, and cellular component were retrieved using the LION/web application (http://www.lipidontology.com/). The LION terms were used for enrichment analysis using a hypergeometric test in DiCoExpress for the lipid species that exhibited differences in their relative abundances among experimental treatments. All data analyses were performed in R 4.0.2 (R Core Team, 2019) using R Studio (RStudio Team, 2020). We used the updated lipid classification proposed by Fahy et al. (2009).

## 3 RESULTS

### 3.1 Data quality and exploratory analysis

**FIGURE 1:**
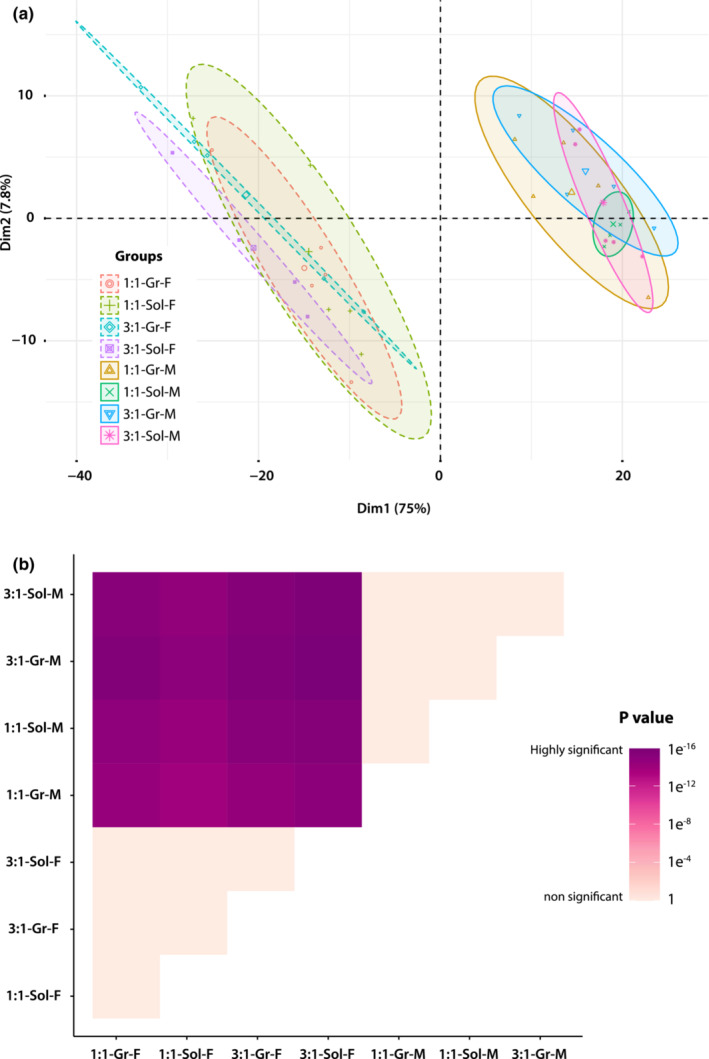
(a) Principal Component Analysis (PCA) of all the biological replicates seen together. Group labels represent the eight experimental treatments resulting from the combination of three factors considered in this study (sex, diet, and social environment. 2 × 2 × 2). (b) Multivariate analysis of variance (MANOVA) including the first three principal components (from the PCA analysis above, which explained 85.95% of variances) as dependent variables. Pairwise comparisons among the eight experimental treatments (i.e., diet × social environment × sex) were conducted using a Wilks' lambda test and Bonferroni correction. For both the PCA and MANOVA, the labels (e.g., 1:1‐Gr‐F) indicate the diet (1:1 or 3:1, protein to carbohydrate ratio), social environment (Sol and Gr, for solitary and group), and sex (F and M, for female and male) for each group of crickets.

**FIGURE 2:**
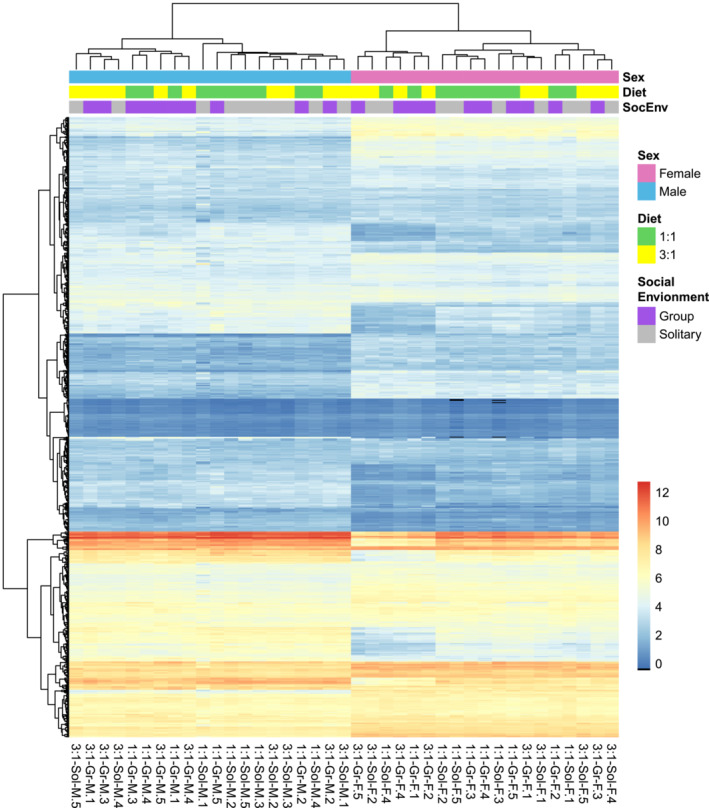
Hierarchical clustering heatmap showing the relative abundance of lipid species and clustering of biological samples according to the experimental treatments (i.e., sex, diet, and social environment). The clustering pattern indicates significant differences between male and female lipidomes, while the other factors do not show a noticeable pattern. The color code on the bottom right corner indicates the log_2_‐transformed lipid area. The labels in the bottom *x*‐axis indicate the sample identity (e.g., cricket 3:1‐Sol‐M.5) by naming the diet (1:1 or 3:1, protein to carbohydrates ratio), social environment (Sol and Gr, for solitary and group), sex (F and M, for female and male), and replicate number (1–5).

The mass spectrometry analysis allowed us to identify 811 distinct lipid species in all insect samples included in this study (complete list of lipid species is available in the Appendix S1). The diagnostic analysis of the samples revealed that there was no batch effect (i.e., unwanted variation) after data normalization (Figure S2 in the Appendix S1), but rather the experimental factors (i.e., sex, diet, and social environment) were driving the clustering of the samples (Figures 1 and 2). However, the exploratory data analysis showed that the experimental treatments did not cluster the samples in an intuitive manner. In the principal component analysis (Figure 1a), MANOVA (*F* = 3.069, *p* < .001) (Figure 1b) as well as in the hierarchical clustering heatmap (Figure 2), it can be seen than the factor “Sex” had a noticeable effect on the grouping pattern, thus suggesting a significantly different lipidome for male and female crickets. Furthermore, the PCA results suggested that there could be differences among female crickets subjected to distinct experimental conditions. However, these differences were not supported by the MANOVA test (Figure 1b). Additionally, the pattern of the samples in the PCA analysis indicates that the variation in the relative lipid quantity in some experimental treatments for both male and female crickets might be substantial. Likewise, a significant variation in the relative abundance of the lipids across treatments is noticeable in the heatmap, with few lipid species being highly abundant. However, this observation is not surprising as in this study, we performed a lipidome‐wide assessment of full‐body insects.

### 3.2 Differences in relative abundance of lipid species

The artificial diets contained a complex mixture of 517 lipids from egg (i.e., the only source of lipids in the ingredients used. For further details on diet composition and recipe, please see Gutiérrez et al. [Gutiérrez, Fresch, et al., 2020, Gutiérrez, Ott, et al., 2020, Gutiérrez, Phung, et al., 2020]). We did not exclude these lipids from the insect dataset as lipid composition and quantity were expected to be the same in both diets (i.e., 3:1 and 1:1) because the recipes contained the same amount of egg powder. Therefore, we do not regard the diets to be a potential source of noise in our dataset.

**FIGURE 3:**
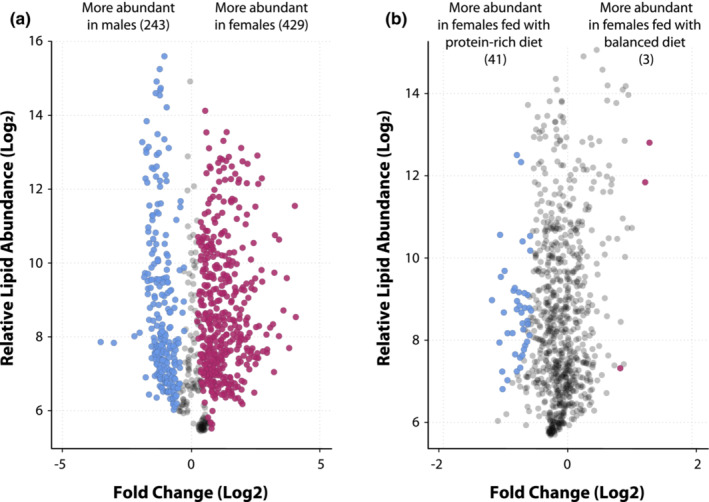
Lipids that exhibited significant differences in their relative abundances when comparing male and female *Acheta domesticus* (a) and only females fed with either the protein‐rich (3:1) or the balanced (1:1) diet (b). For a complete list of lipids and their corresponding FDR values, please see Tables S5 and S6 in the Appendix S1. Relative abundances as lipid fragment ion peak areas (in the *y* axis) were transformed to Log_2_ scale.

The relative abundances of a considerable number of lipid species in the crickets *A. domesticus* were significantly changed by some of the factors included in our experiment. On the one hand, sex was the most influential factor in the lipidome profile of house crickets (Figure 3a). Male crickets had a higher abundance of 243 lipids (29.97%), while 249 lipid species (30.70%) were more abundant in female *A. domesticus* (see complete lipid list and statistical results in Table S5 in the Appendix S1). Due to this striking difference between sexes, we studied the effects of diet and social environment on the relative abundance of lipid species for male and female crickets separately to achieve a more precise interpretation of the results.

**FIGURE 4:**
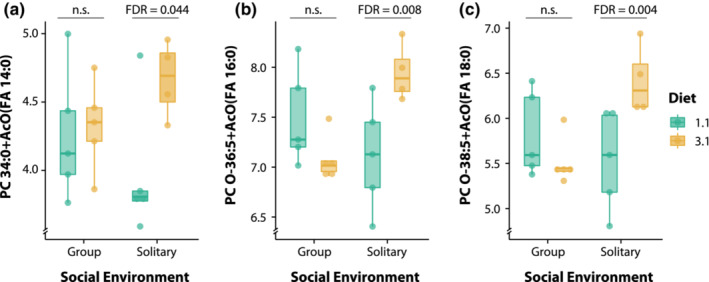
Changes in lipid relative abundance induced by diet composition and social environment in female *Acheta domesticus* in a two‐way interaction. The abundance of PC 34:0 + AcO(FA 14:0) (a), PC O‐36:5 + AcO(FA 16:0) (b), and PC O‐38:5 + AcO(FA 18:0) (c) was higher in females living solitarily and fed with the protein‐rich diet (3:1). Relative abundance as lipid fragment ion peak areas (in the *y* axis) was transformed to Log_2_ scale. FDR stands for False Discovery Rate, a method for adjusting *p*‐values and controlling type I errors.

Diet composition led to interesting lipidome profile changes. Female crickets exhibited a higher abundance of 41 lipid species (5.05%) when fed with a protein‐rich diet, while their counterparts fed with the balanced diet exhibited only three lipids with higher abundances (Figure 3b, see complete lipid list and statistical results in Table S6 in the Appendix S1). Furthermore, the interaction of diet composition and the social environment induced a greater abundance of the lipid species PC 34:0 + AcO(FA 14:0) (fold‐change Log_2_ = 0.96, Likelihood ratio = 10.58, FDR = 0.044, Figure 4a), PC O‐36:5 + AcO(FA 16:0) (fold‐change Log_2_ = 1.12, Likelihood ratio = 15.11, FDR = 0.008, Figure 4b), and PC O‐38:5 + AcO(FA 18:0) (fold‐change Log_2_ = 1.16, Likelihood ratio = 16.86, FDR = 0.004, Figure 4c) only when females living in solitarity were fed with the protein‐rich diet. Interestingly, the lipidome of male crickets was unresponsive to the experimental conditions imposed through the manipulation of their diet composition and social environment.

### 3.3 Functional analysis

Using the lipid ontology tool LION/web (Molenaar et al., 2019), we identified 3947 LION terms associated with our lipid species list (all data are available in the Appendix S1). From these, 2149 terms were associated with lipid classification, 985 with cellular components, and 813 with biological functions.

**FIGURE 5:**
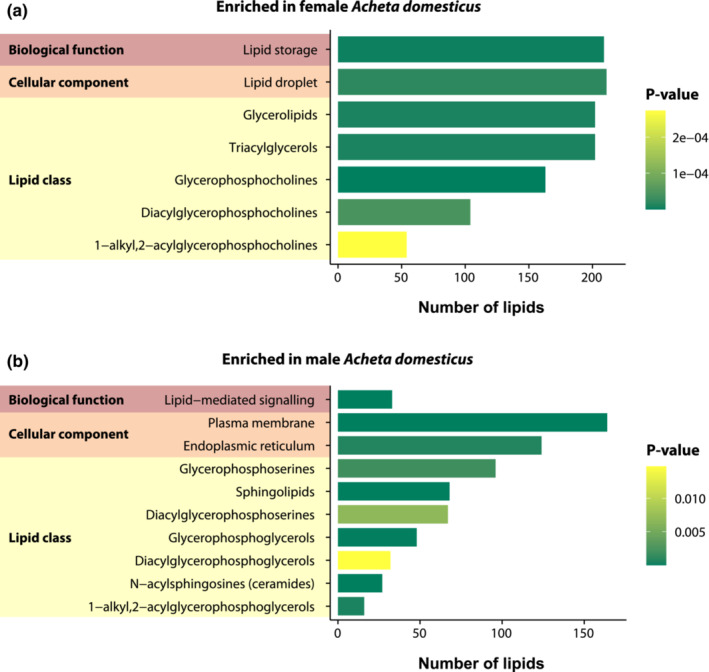
Enrichment analysis for LION (Lipid Ontology) terms associated with differentially abundant lipids due to sex‐related differences in female (a) and male (b) *Acheta domesticus*. For further details, please see Table S7 in the Appendix S1.

First, we conducted an enrichment analysis focusing on the lipid species that had their relative abundances significantly changed due to sex only (Figure 5). In this case, female crickets had an overrepresentation of lipid storage, which implies that highly abundant lipids in females were probably related with this biological function. Accordingly, the cellular component with significant enrichment in female crickets was the lipid droplets. Besides, the lipid classes overrepresented in female crickets were the glycerophosphocholines [GP01] (with the subclasses diacylglycerophosphocholines [GP0101] and 1‐alkyl,2‐acylglycerophosphocholines [GP0102]); and the category glycerolipids [GL] (with the subclass triacylglycerols [GL0301]) (Figure 5a). On the other hand, male crickets had their lipid‐mediated signaling biological function significantly enriched. The cellular components with an overrepresentation of lipid species were the plasma membrane and the endoplasmic reticulum. In addition, the lipid classes enriched in male *A. domesticus* were the category sphingolipids [SP] (with the subclass N‐acylsphingosines (ceramides) [SP0201]), the class glycerophosphoserines [GP03] (with the subclass diacylglycerophosphoserines [GP0301]), and the class glycerophosphoglycerols [GP04] (with the subclasses diacylglycerophosphoglycerols [GP0401] and 1‐alkyl,2‐acylglycerophosphoglycerols [GP0402]) (Figure 5b. The complete results of the enrichment analysis for both sexes are available in Table S7 in the Appendix S1).

**FIGURE 6:**
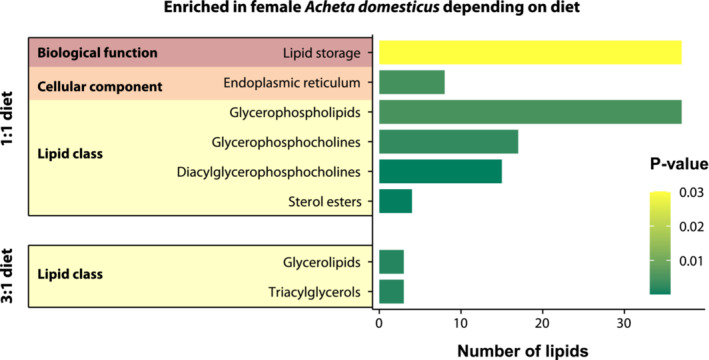
Enrichment analysis for LION (Lipid Ontology) terms associated with differentially abundant lipids in female *A. domesticus* fed with diets of different composition (i.e., protein‐rich [3:1] or balanced [1:1]). For further details, please see Table S8 in the Appendix S1.

Second, an additional enrichment analysis was performed using the set of lipids with differential abundance in female *A. domesticus* fed with diets of different composition. Female crickets fed with the balanced diet had their lipid storage function enriched, and the endoplasmic reticulum was the cellular component with overrepresentation of lipids. Furthermore, the lipid class enriched was the category glycerophospholipids [GP] (with the class glycerophosphocholines [GP01] and the subclass diacylglycerophosphocholines [GP0101]) and the subclass sterol esters [ST0102]. On the contrary, female crickets fed with the protein‐rich diet only had a significant overrepresentation of the category glycerolipids [GL], with the subclass triacylglycerols [GL0301]. No biological function or cellular component was significantly enriched in females fed with the balanced diet. (Figure 6, the complete results of the enrichment analysis for females subjected to either of both diets are available in Table S8 in the Appendix S1).

We did not perform an enrichment analysis for the lipids that exhibited significant differences in response to the interaction of the factors diet composition and social environment as the list was too short (i.e., only three lipid species were more abundant when females were housed in solitarity and fed with the protein‐rich diet). In such case, all three lipids belong to the category glycerophospholipids [GP]. The lipids PC O‐36:5 + AcO(FA 16:0), and PC O‐38:5 + AcO(FA 18:0) are included in the class glycerophosphocholines [GP01] (within the subclass 1‐alkyl,2‐acylglycerophosphocholines [GP0102]), and the lipid PC 34:0 + AcO(FA 14:0) is included in the class glycerophosphocholines [GP01] (within the subclass diacylglycerophosphocholines [GP0101]). All three aforementioned lipids with significantly higher abundance in female crickets fed with the balanced diet are components of the membrane and are located in the endoplasmic reticulum according with their functional annotation.

## 4 DISCUSSION

In this study, we performed a lipidome‐wide assessment of the house‐cricket *A. domesticus* when individuals were subjected to distinct experimental conditions differing in the diet constitution and social environment. We used high‐throughput MS‐based techniques for data acquisition, a recently developed framework for multifactorial analysis of ‘omics’ datasets (Lambert et al., 2020), and a novel database for lipid ontology annotation (Molenaar et al., 2019). Overall, we found that sex had a striking effect on the lipid profile of the crickets. Furthermore, females were highly responsive to diet constitution, while males remained unaffected. In addition, the social environment did not induce significant changes in the relative abundance of lipid species and their associated functions in the house cricket *A. domesticus*.

In our previous study, we demonstrated that adult female crickets have an overall higher lipid content than adult males (Gutiérrez, Fresch, et al., 2020), which is suspected to be related with the storage of lipids for embryogenesis (Cripps et al., 1988). The results obtained in this study allow us to conclude that such difference in lipid composition of male and female *A. domesticus* is not only in terms of total abundance but also in the relative abundance of particular lipids with specific functions in these insects. About a third of the identified lipid species in these crickets were significantly different between males and females, and our functional analysis revealed that most of these differences are related with the sex‐specific life strategy of these animals. The sex‐related difference in the lipidome has already been reported in species such as fruit‐fly *Drosophila* and in humans (Mertens et al., 2017; Scheitz et al., 2013).

On the one hand, the lipidome of female crickets is highly tailored toward intracellular storage of energy in lipid droplets, which is readily available according to metabolic needs (Ducharme & Bickel, 2008). These organelles are found in several taxa ranging from bacteria to mammals and play an important role in the synthesis, utilization, and mobilization of lipids (Suzuki et al., 2011). These organelles can be found in almost any type of cell within an organism; yet, they are highly abundant in insect eggs (i.e., underneath the oocyte cortex) (Van Antwerpen et al., 2005; Ziegler & Van Antwerpen, 2006). Therefore, we infer that the enrichment of lipid storage and particularly, lipid droplets, suggests that the more abundant glycerolipids and glycerophosphocholines in adult female crickets were mainly intended for vitellogenesis (i.e., yolk accumulation in the oocytes). On the other hand, the differentially abundant lipid species in male crickets are mainly related with signaling processes, which are responsible for homeostasis at the cell and organismal level (Owusu‐Ansah & Perrimon, 2014; Palm et al., 2012). Such signaling processes overrepresented in male crickets indicate that these have a relatively higher investment in processes like regulation of cell proliferation, migration, and apoptosis than female *A. domesticus* (Hannun & Obeid, 2008; Herr et al., 2003; Watson, 2006). Although the previously mentioned processes act mainly at the cellular level, they would escalate to organismal processes such as muscle development and reproductive output (Herr et al., 2003; Prieschl & Baumruker, 2000).

Even though female crickets appear to heavily invest in lipid storage as a life‐strategy mechanism, according to our results, female *A. domesticus* fed with the balanced diet seems to further emphasize in this strategy. This finding contradicts our initial hypothesis, as we expected to find a higher abundance of storage lipids in females fed with the protein‐rich diet to fuel their higher fecundity (Gutiérrez, Fresch, et al., 2020; Ziegler & Van Antwerpen, 2006). A larger lipid storage in female *A. domesticus* fed with the balanced diet is likely derived from excess of carbohydrates available (i.e., 1:1, protein to carbohydrates). This larger energy reserve may be related with the longer lifespan recorded in female *A. domesticus* fed with the balanced diet but certainly does not explain their lower fecundity (Gutiérrez, Fresch, et al., 2020). It is then possible than a lower lipid storage in female crickets fed with the protein‐rich diet is not directly related with their higher fecundity, but it would be related with their shorter lifespan. As previous studies have shown, a lipid reserve is paramount in the survival of many organisms due to the tight association of energy homeostasis and lipid metabolism (Gutiérrez, Phung, et al., 2020; Wenk, 2010). In addition, the excess nitrogen in the protein‐rich diet could be detrimental for female crickets due to nitrogen toxicity reported in other insect species (Cook et al., 2010; Dussutour & Simpson, 2012). Also in this context, the lower lipid reserves in male crickets (evidenced in this study) and the overall higher metabolism may partially explain the relatively shorter lifespan of male *A. domesticus* than that of females (Gutiérrez et al., 2021; Visanuvimol & Bertram, 2011).

Taken together, our results suggest that excess of dietary protein would be the primary driver of higher fecundity, while the ability to store lipids derived from a diet richer in carbohydrates would confer a longer lifespan in female *A. domesticus*. Furthermore, it is possible that the absence of the effect of diet composition on male crickets and social environment in male and female crickets is due to a substantial intra‐treatment variation (as noticed in our initial exploratory analysis). Yet, the high heterogeneity in the relative abundance of some lipid species cannot be explained by technical variation as a highly complex internal standard blend was used in this study for data normalization, and we did not detect batch effects. Therefore, such heterogeneity can be rather attributed to biological variation among samples of the same treatment. Even though crickets were age‐standardized and subjected to highly controlled experimental conditions, individual‐specific responses can be expected (Assfalg et al., 2008; Wenk, 2010). Furthermore, although the genetic background of the crickets used in this study is unknown, commercially produced cricket populations are known for their low genetic diversity induced by inbreeding depression and genetic bottleneck (Gupta et al., 2020). Conducting these types of studies with a single (or at least more restricted) genetic background might provide insights into biological pathways by reducing the biological variation (Chow, 2016). Conversely, more generalizability at the eco‐physiological level can be accomplished by incorporating a more diverse genetic background into the experimental design and increasing the sample size (Sittig et al., 2016).

By analyzing whole‐body insects, we gained insight into the full picture of the metabolism of this organism. Yet, this approach might have obscured differences specific to certain organs and tissues as a big portion lipidome tends to be organ‐specific (Carvalho et al., 2012). Moreover, it is worth considering that the altered biological functions identified through the functional analysis might overlook processes potentially related with the changes in the lipidome profile as the dataset used is still in development (Molenaar et al., 2019). Here, the changes in the lipidome were mainly related with lipid storage and lipid‐mediated signaling only, while several studies have shown that lipids can be useful biomarkers for diseases of different nature involving several physiological mechanisms (Ertunc & Hotamisligil, 2016; Wang et al., 2016; Wenk, 2010). Although many tools for lipidomic analysis and annotation are becoming increasingly available, most of them still lack straightforward inference of biological function enrichment or are oriented toward clinical sciences or particular model organisms (e.g., LIPEA, LipidPedia, SwissLipids). Another matter worth considering in future studies is the selection of internal standards when performing a lipidome analysis. In this study, we used the commercially available EquiSPLASH™ Lipidomix® (Avanti Polar Lipids), which includes 13 deuterium‐labeled lipids, one for every major lipid class. However, this internal standard did not include a standard for ether lipids, a class of glycerophospholipids occurring in insects, as shown in our dataset. Therefore, the quantification accuracy of certain lipids may be increased by including further internal standards according to the nature of the samples.

All considered, our results suggest that the growing conditions, particularly diet composition, may have a significant impact on the lipidome of the cricket *A. domesticus*, and consequently, on its physiology. However, the response to the experimental conditions to which insects are subjected can be highly sex‐specific, as highlighted in several past studies, urging the need to address ecological and physiological questions in a sex‐specific manner (Goos et al., 2016; Maklakov et al., 2008; Morehouse et al., 2010). Here, the lipidome of female crickets was significantly influenced by the composition of the diet, thus potentially having an impact on its fecundity and lifespan. Contrarily, the lipidome of male crickets appears to be more robust as no significant difference was noticed across experimental treatments.

## AUTHOR CONTRIBUTIONS

**Yeisson Gutiérrez:** Conceptualization (lead); data curation (equal); formal analysis (lead); methodology (equal); writing – original draft (lead); writing – review and editing (lead). **Marion Fresch:** Data curation (equal); methodology (equal); writing – review and editing (equal). **Christoph Scherber:** Conceptualization (equal); formal analysis (equal); funding acquisition (equal); supervision (equal); writing – review and editing (equal). **Jens Brockmeyer:** Conceptualization (equal); funding acquisition (lead); methodology (equal); supervision (equal); writing – original draft (equal).

## CONFLICT OF INTEREST

The authors declare that they have no competing interests.

## Supporting information


Appendix S1

## Data Availability

All data analysed in this study are included in the supplementary material and the R scripts are available at Dryad (https://doi.org/10.5061/dryad.hqbzkh1jc).
