# Supplementary material for: The lipidome of an omnivorous insect responds to diet composition and social environment
Source: Ecol Evol. 2022 Nov 8;12(11):e9497. doi: 10.1002/ece3.9497 (PMC9643132; doi:10.1002/ece3.9497)
Supplement: Supplementary file 1 — Appendix S1 [file ECE3-12-e9497-s001.zip › ECE3_9497_Supplementary Material.pdf]

Inferring physiological changes induced by diet composition and social environment at the organism-level through lipidomics analysis using an omnivorous insect as a model organism

Yeisson Gutiérrez, Marion Fresch, Christoph Scherber, Jens Brockmeyer

NOTE: All tables are included in a separate Excel file to grant access to all data in a handy format

Table. S1. Mixture of deuterium-labelled lipids covering all lipid classes used as internal standards in this study for peak area normalization. EquiSPLASH™ LIPIDOMIX® Quantitative Mass Spec Internal Standard (Avanti Polar Lipids, Inc., USA)

Table S2. Operation conditions for FIA-MS/MS quantitation of lipids

Table S3. Analysis method properties for lipid identification in both positive and negative ion modes. Deisotope refers to the removal of isotope contribution in lipid species with close lower m/z.

Table S4. Definition of internal standards. Precursor and fragment ions of each internal standard used. EquiSPLASH™ LIPIDOMIX® Quantitative Mass Spec Internal Standard (Avanti Polar Lipids, Inc., USA)

Table S5. Differential relative abundance results for lipid species affected by the factor "sex". Positive fold-change indicates higher abundance of lipid species in females and negative values indicate higher abundance in male crickets

Table S6. Differential relative abundance results for lipid species affected by diet composition in female *Acheta domesticus*. Positive fold-change indicates higher abundance of lipid species when females were fed with the balanced diet (1:1) and negative values indicate higher abundance when females received the protein-rich diet (3:1)

Table S7. Complete results for the enrichment analysis. LION (Lipid Ontology) terms overrepresented in male and female crickets. BP: Biological Process. MF: Molecular Function. CC: Cellular Component

Table S8. Complete results for the enrichment analysis. LION (Lipid Ontology) terms overrepresented in female crickets in response to diet composition. BP: Biological Process. MF: Molecular Function. CC: Cellular Component

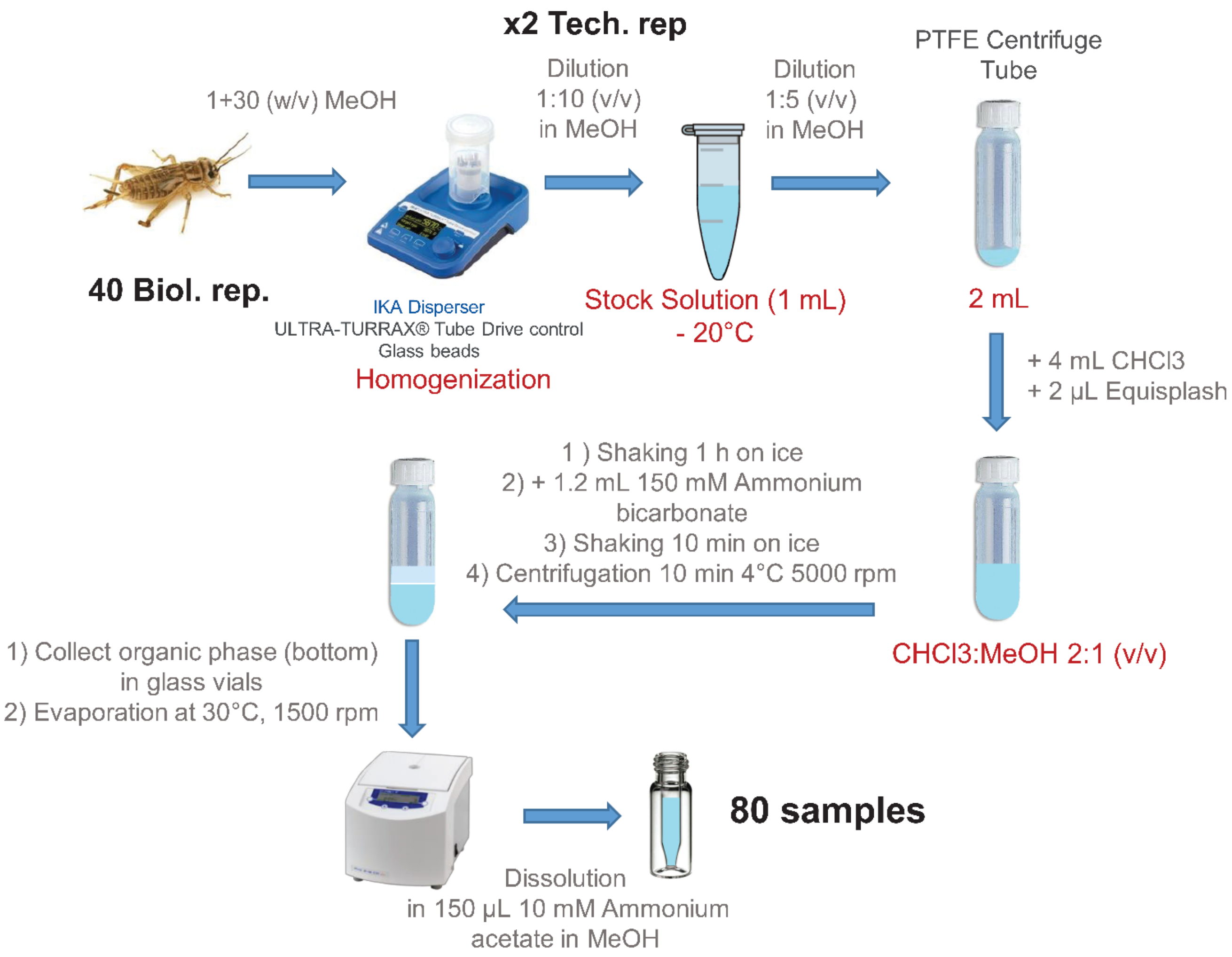

Fig. S1. Summary of the lipid extraction protocol performed in this study

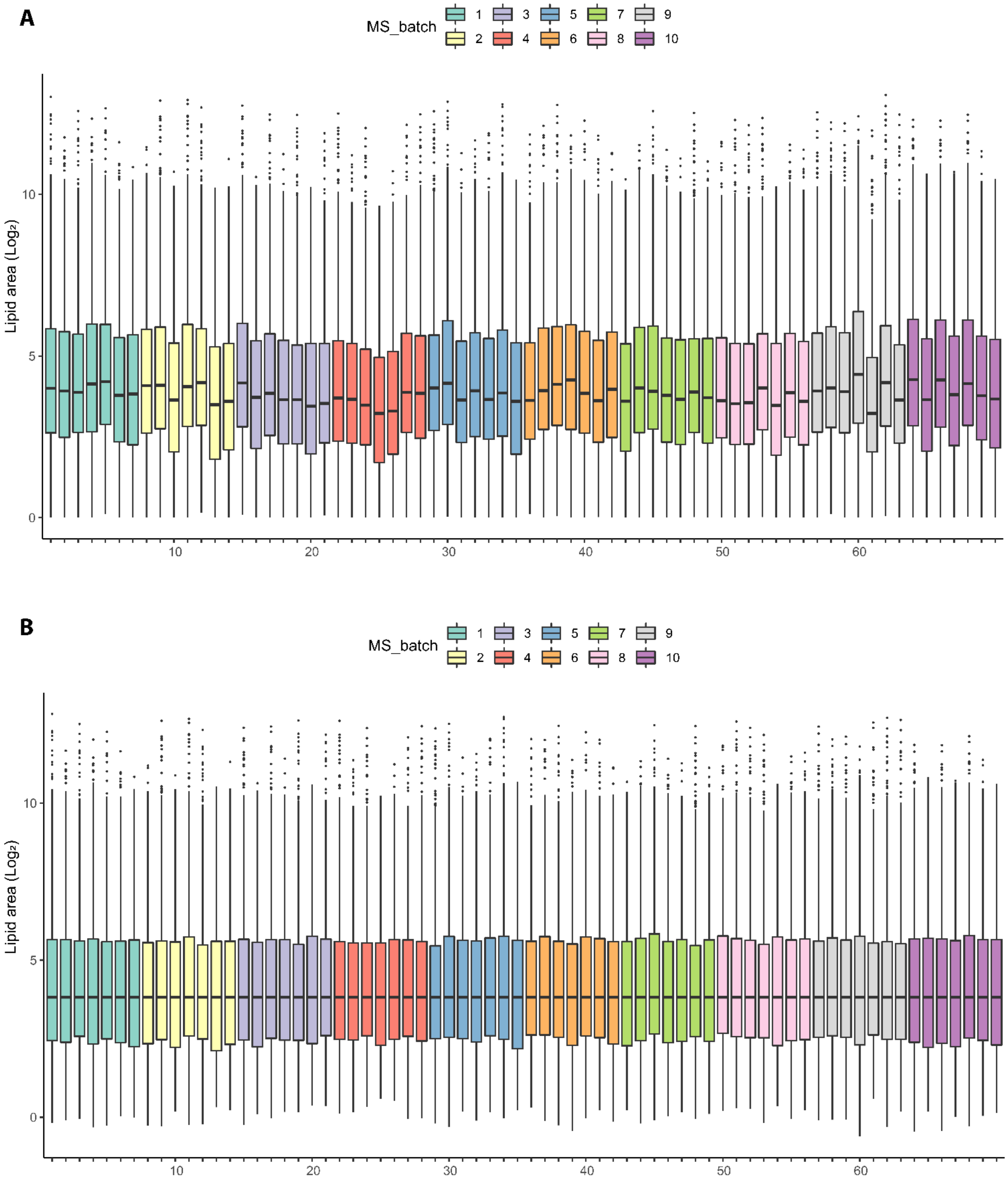

Fig. S2. Peak area of the lipid species for all technical replicates. Data was normalized by using internal standards (see Tables S1 and S4) and Log2-transformed (A). Posteriorly, the sample medians were centred to the global median of the data (B). Colours indicate the batches in which lipid data was acquired between calibration runs.
